# Variations of the NodB Architecture Are Attuned to Functional Specificities into and beyond the Carbohydrate Esterase Family 4

**DOI:** 10.3390/biom14030325

**Published:** 2024-03-08

**Authors:** Alexis S. Molfetas, Nikiforos Boutris, Anastasia Tomatsidou, Michael Kokkinidis, Vasiliki E. Fadouloglou

**Affiliations:** 1 Institute of Molecular Biology and Biotechnology, 70013 Heraklion, Crete, Greece; asmolf@gmail.com (A.S.M.); mikekok@otenet.gr (M.K.); 2 Department of Biology, University of Crete, Voutes University Campus, 70013 Heraklion, Crete, Greece; tomatsidou@uchicago.edu; 3 Department of Molecular Biology and Genetics, Democritus University of Thrace, Dragana University Campus, 68100 Alexandroupolis, Evros, Greece; nickboutris@gmail.com

**Keywords:** distorted TIM-barrel, polysaccharide, peptidoglycan, biofilm, carbohydrate esterases, PuuE allantoinases

## Abstract

Enzymes of the carbohydrate esterase family 4 (CE4) deacetylate a broad range of substrates, including linear, branched and mesh-like polysaccharides. Although they are enzymes of variable amino acid sequence length, they all comprise the conserved catalytic domain NodB. NodB carries the metal binding and active site residues and is characterized by a set of conserved sequence motifs, which are linked to the deacetylation activity. Besides a non-structured, flexible peptide of variable length that precedes NodB, several members of the CE4 family contain additional domains whose function or contribution to substrate specificity are not efficiently characterized. Evidence suggests that CE4 family members comprising solely the NodB domain have developed features linked to a variety of substrate specificities. To understand the NodB-based substrate diversity within the CE4 family, we perform a comparative analysis of all NodB domains structurally characterized so far. We show that amino acid sequence variations, topology diversities and excursions away from the framework structure give rise to different NodB domain classes associated with different substrate specificities and particular functions within and beyond the CE4 family. Our work reveals a link between specific NodB domain characteristics and substrate recognition. Thus, the details of the fold are clarified, and the structural basis of its variations is deciphered and associated with function. The conclusions of this work are also used to make predictions and propose specific functions for biochemically/enzymatically uncharacterized NodB-containing proteins, which have generally been considered as putative CE4 deacetylases. We show that some of them probably belong to different enzymatic families.

## 1. Introduction

**Figure 1:**
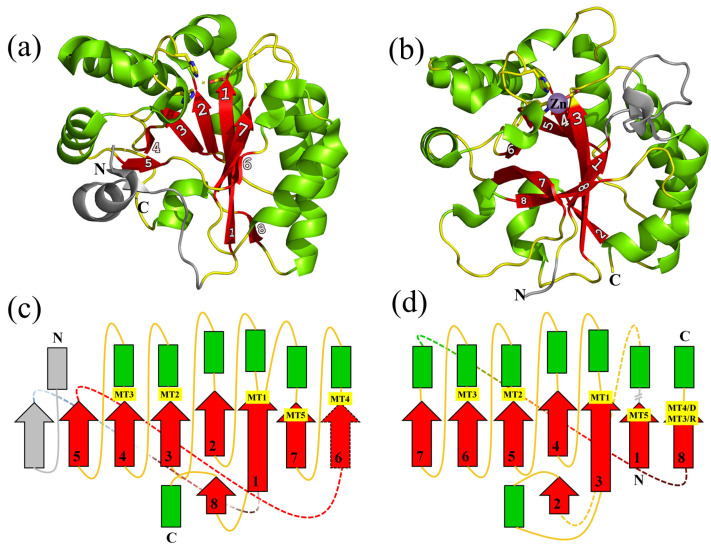
NodB 3D structure, topology and motif (MT) distribution. The NodB homology domain is represented by the structures of (**a**) Bc1960, PDB id 4L1G and (**b**) the C-terminal domain (CTD) of Bc0361, PDB id 4HD5. The topological diagrams of Bc1960 (**c**) and CTD of Bc0361 (**d**) are representative of the NodB1 and NodB2 topologies, respectively. The residues of the metal-binding triad are shown by stick representation on panels (**a**,**b**). Although the structure of the Bc1960 (PDB id 4L1G) has been solved without metal, the cross mark in panel (**a**) indicates the expected metal-binding position. On the other hand, the zinc is represented by a purple sphere in panel (**b**). In the topological diagrams, the NodB β-strands are numbered sequentially from the N- to the C-terminus. Secondary structure elements are connected by lines. Different line colors and line styles are used for clarity. The framework NodB structure elements are colored green (α-helices) and red (β-strands). Additional parts of the sequence are shown in grey. Especially, the grey ‘long loop and half-turn helix’ element of (**b**) panel is represented by the symbol ‘-//-’ between strand 1 and the following helix in the corresponding (**d**) panel. The positions of conserved motifs (MT1-5) are also shown in the topological diagrams.

The β/α, TIM-barrel (triose-phosphate isomerase) structures are the most frequently occurring folding motifs in proteins [1,2]. Their framework structure comprises approx. 150–200 residues, which are folded in a tandem repetition of β/α units, resulting in a central, parallel β-barrel surrounded by an equal number of α-helices. The structure has two hydrophobic interfaces, an inner barrel core and an outer barrel layer formed between the β-strands and the α-helices. β/α barrel enzymes have their active sites consistently located at the carboxyl termini of the β-strands, especially on the loops that link the β-strands with the succeeding α-helices (βα loops). βα loops present high variability among the fold and often incorporate peptides of different lengths, sequences and conformations. Entire domains can protrude from these loops, providing structural variability that enables the framework structure to accommodate a wide range of catalytic activities in a variety of substrates [3,4]. In addition to the highly regular (β/α)_8_ TIM variant, several other variants of β/α barrels exist. The NodB domain is a distorted, TIM-like barrel (Figure 1a,b), classified in the c.6.2.3 and c.6.2.6 families of the c.6.2 superfamily of glycoside hydrolase/deacetylase in the SCOPe-2.08 database (pfam 01522) [5,6]. As expected, the active site is formed at the C-termini of the β-strands, incorporating residues from the βα loops and containing a metal ion, usually zinc, which is coordinated by a His-His-Asp triad [7]. As shown in other cases, the barrel’s distortion creates a crevice that accommodates at least part of the substrate (Figure 1). Five conserved sequence motifs (MT1 to MT5) play key roles in the catalytic activity of the NodB domain [7,8]. They incorporate the catalytic residues, the metal binding site, and an electron relay pair of residues, as well as a highly conserved proline residue of critical importance due to its ability to be post-translationally modified to 2-hydroxyproline (2-Hyp) [9,10,11].

The NodB domain has originally been recognized and studied as the homologous, catalytic domain of the carbohydrate esterases of family 4 (CE4), which deacetylates polysaccharides [12,13]. The NodB domain was named after the Rhizobium NodB protein, the first member of the CE4 family to be characterized [14]. This protein is involved in the synthesis of a lipo-oligosaccharide signal molecule by deacetylating the non-reducing GlcNAc residue of chito-oligosaccharides. The deacetylase activity of the CE4 family (CAZy database [15]; http://www.cazy.org, accessed on 1 July 2023) resides exclusively on the NodB domain. CE4 catalyzes the deacetylation reaction through *N*-acetyl and *O*-acetyl esteric-bond hydrolysis on a broad range of substituted carbohydrates. Compared to other carbohydrate esterases, family 4 is by far the largest, incorporating sequences from Gram-positive and Gram-negative bacteria, archaea, fungi and viruses [12]. Furthermore, the CE4 family shows the second largest substrate diversity among the CE families, acting on at least five classes of polysaccharides, i.e., chitin and chito-oligosaccharides (COS), poly-β-1,6 *N*-acetyl-*D*-glucosamine (PNAG), xylan and peptidoglycan (PG), with clear discrimination between acetylglucosamine and acetylmuramic acid moieties. Chitin, COS and PNAG are linear polymers purely composed of *N*-acetylglucosamine units (GlcNAc), which are linked by β-(1→4) glycosidic bonds in chitin/COS and β-(1→6) glycosidic bonds in PNAG. Xylans are branched polysaccharides composed of xylopyranosyl residues linked together by β-(1→4) and/or β-(1→3) glycosidic bonds. PG, however, forms layers and is composed of the disaccharide *N*-acetylglucosamine/*N*-acetylmuramic acid (GlcNAc/MurNAc), where the sugar units are linked together by β-(1→4) glycosidic bonds. Attached to the MurNAc unit is a short peptide of three to five residues depending on the bacterium, which can be cross-linked with the peptide of another sugar strand, forming a layered structure [8].

Although additional domains are present in several CE4 family members, many other members associated with diverse substrate specificities consist entirely of a NodB domain. This observation implies that NodB has all the necessary information not only for catalytic activity but also for substrate-specific recognition and binding. This feature can be exemplified by examining two proteins that both comprise a single-NodB domain, the 275-residue-long *Bacillus cereus* Bc1960 and the 286-residue-long *Clostridium thermocellum* CtCE4 [9,16]. The first shows a PG GlcNAc de-N-acetylase specificity, while the second is a de-O-acetylxylan esterase. Additionally, the 445-residue-long *Streptococcus pneumonia* SpPgdA protein [7], which is composed of three different domains, including the NodB, is also a PG GlcNAc de-N-acetylase similar to Bc1960. Therefore, additional domains are not conclusively related to substrate specificity and binding. Given that the single-NodB-domain enzymes exhibit different substrate specificities, it seems reasonable to hypothesize that apart from substrate processing, NodB plays a critical role in substrate recognition and binding. Thus, the current hypothesis is that sequence, topology and structural excursion variations on the framework structure of this domain give rise to variations in substrate specificity for different members of the family and beyond the family. Indeed, there is extended recent literature that implies that in analogy with the canonical TIM-barrels, the variations in the βα loops must be related to substrate specificity [17,18,19,20]. In addition to length, sequence and conformation, dynamics and plasticity of the loops seem to play a role in the adaptivity of the binding site to the different substrates and, consequently, to the specificity [18]. Interestingly, according to recent literature, the NodB domain is present in newly identified enzyme families, i.e., CE18 and an atypical PuuE-like allantoinase [21,22]. 

Here, we analyze the sequences, sequence relationships, topology, and 3D architectural variations in the space of CE4 NodB domains with determined 3D structures. We identify and describe features potentially associated with substrate and functional diversity and subsequently categorize the NodB barrels into classes. The analysis is combined with biochemical and enzymatic data available in the literature in an attempt to understand the basis of the catalytic specificity and make the structure-function corresponding analysis where possible. Our dataset consists of all the NodB domains found in proteins identified as CE4 enzymes or characterized as putative CE4 deacetylases (CAZy database, http://www.cazy.org; accessed on 1 July 2023 [15]) with known 3D structures (Table S1). Our findings are compared with NodB domains belonging to newly characterized enzymatic families. We show that conserved sequence motifs, in combination with sequence variability and structural plasticity, are consistent with a common catalytic mechanism and a remarkable diversity in polysaccharide substrate specificities. Sequence motifs that have been thus far overlooked emerge from our analysis, and most importantly, two distinct topologies forming the same barrel architecture point to putatively distinct evolutionary origins and pave the way for improved predictions of functionalities within and beyond the CE4 family. Indeed, based on the conclusions drawn from our analysis, we propose functions for as-yet uncharacterized proteins. 

## 2. Materials and Methods

### 2.1. Dataset Construction

The psi-BLAST search program through ExPASy BLAST server interface (https://blast.ncbi.nlm.nih.gov/Blast.cgi?PAGE=Proteins; accessed on 1 July 2023) was used to obtain a list of all NodB domain-containing proteins with determined three-dimensional structures. The results were cross-checked with the PDB matches to PROSITE database entry PS51677 (NodB homology domain; https://prosite.expasy.org/; accessed on 1 July 2023), the SCOPe database (c.6.2.3 and c.6.2.6; https://scop.berkeley.edu/; accessed on 1 July 2023), and the pfam PF01522 of InterPro database (https://www.ebi.ac.uk/interpro/; accessed on 1 July 2023) and further expanded by an extensive and meticulous search of the related literature. The complete final list used in this study is shown in Table S1 and contains proteins from Gram-positive and Gram-negative bacteria, fungi and insects. 

### 2.2. Clustering Analysis

**Figure 2:**
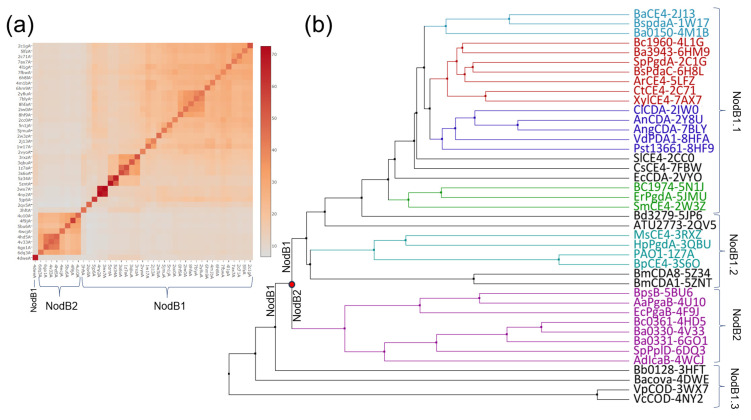
Structural and clustering analysis of the NodB domain (Table S1). (**a**) Heatmap of structural similarity matrix based on Dali Z-scores. (**b**) An average distance dendrogram that was determined by a structure-aided multiple sequence alignment using PAM250 (see Section 2).

A multiple sequence structure-aided alignment of the NodB domains was carried out through T-Coffee/Expresso [23]. Low complexity and poorly aligned regions were removed from the alignment manually. A substantial number of dendrograms were generated by Jalview using different parameters [24]. It was verified that all these trees share a common topology. Figure 2b presents an average distance dendrogram constructed using the PAM250 substitution matrix.

### 2.3. Topology Analysis

Topological diagrams were constructed manually by visual inspection of the individual structures.

### 2.4. Correspondence Analysis

An all-against-all structural comparison of our sample was performed by the Dali server (http://ekhidna2.biocenter.helsinki.fi/dali/, accessed on 1 August 2023 [25]), and it is presented as a heatmap in Figure 2a. 

### 2.5. Function-Related Sequence Conservations Mapped on the NodB Fold

Four multiple sequence alignments, including proteins of confirmed function, i.e., GlucNAc PG deacetylase (DA), MurNAc PG DA, chitin/COS DA and PNAG DA (Table S2), were used for function-related sequence conservation analysis. For each of the four functional groups, a representative structure has been used to present sequence conservations of the family. The color code ranges from dark blue (non-conserved) to red (highly conserved) through to white. The representative structures are colored by sequence conservation scores using ChimeraX [26]. 

## 3. Results

### 3.1. Structural Classification and Clustering Analysis of the NodB Domain

An all-against-all structure comparison of the experimentally determined NodB folds (CE4/NodB domains of known 3D structure, Table S1) was performed by the DALI server. The analysis divided the dataset into two distinct classes designated NodB1 and NodB2 (Figure 2a and Figure S1). NodB1 class is the most populated and includes all but 6DQ3, 6GO1, 4V33, 4HD5, 4WCJ, 5BU6, 4F9J and 4U10 proteins (PDB ids) [9,27,28,29,30,31,32]. These two broad classes are not homogeneous and can be further divided into smaller groups. In Figure 2a, the pairs of 4NY2/3WX7, 5ZNT/5Z34, 3S6O/1Z7A and 1W17/2J13 [17,33,34,35,36] are distinguished as close relatives. The following groups are clearly separated: 8HF9, 2IW0, 8HFA, 7BLY, 2Y8U (shown in blue in the average distance dendrogram presented in Figure 2b) and 5ZNT, 5Z34, 3S6O, 1Z7A, 3QBU and 3RXZ proteins (shown in black/turquoise in Figure 2b). 

The structural similarity patterns identified by DALI (Figure 2a) were confirmed and further documented by a clustering analysis based on a multiple sequence alignment using structural information (Figure 2b). The analysis reveals the presence of two distinct evolutionary groups and suggests an early separation of NodB1/NodB2 domains. Interestingly, the NodB1 domains are classified into groups that broadly coincide with the functional families determined by experimental approaches and are presented in Table S1. For instance, most of the proteins shown in blue (Figure 2b) are chitin/COS deacetylases (DAs). The functionally uncharacterized 3RXZ, 3QBU, 1Z7A and 3S6O (turquoise), together with the more distant insect chitin/COS DAs 5ZNT and 5Z34 (black, [36,37]), constitute another subclass in the tree (Figure 2b). Interestingly, the acetylxylan esterases 5LFZ, 2C71 and 7AX7 are grouped together with the GlcNAc PG DAs 4L1G, 2C1G, the dual PG/chitin DA 6H8L [38] and the uncharacterized 6HM9 (shown in red in the average distance dendrogram). The MurNAc PG DAs 2J13 and 1W17 are grouped together with the pseudoenzyme 4M1B (cyan), implying that 4M1B evolved from MurNAc DAs. Likewise, the PG DA 5N1J [39] constitutes a group with the uncharacterized 5JMU and 2W3Z (green). The NodB2 domain (the purple group of the tree) comprises the uncharacterized Bc0361/Ba0330 proteins as well as the PNAG DAs, implying that this pair of proteins might belong to the specific functional group (Figure 2b). These findings suggest that the NodB domain carries—beyond catalytic properties—additional information linked to substrate specificity. 

### 3.2. Topology Analysis

**Figure 3:**
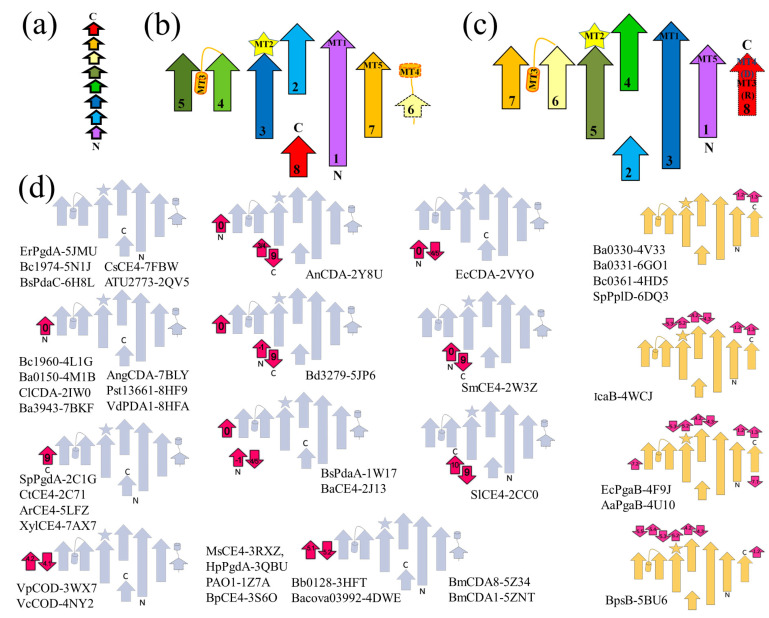
Topological analysis of the NodB architecture. (**b**,**c**) represent the NodB1 and NodB2 topologies, respectively, while (**a**) shows the color scheme of sequential order used in (**b**,**c**). For the sake of simplicity, only the β-strand arrangement is shown. (**d**) Classification of the dataset into NodB1 (grey) and NodB2 (yellow) topologies (for strands’ direction and numbering see panels (**b**,**c**), respectively). The core NodB topology is decorated by additional elements (shown in magenta) that are either incorporated on the termini or inserted within the domain sequence. Their numbering follows that of the core topology shown in panels (**b**,**c**) for each group of topologies. The additional elements confer significant structural variability to the domain, and based on them, members of each main topology are further divided into subgroups.

Further analysis identifies a fundamental topological difference between the NodB1 and NodB2 variations, which in Figure 1 are exemplified by the topologies of enzymes Bc1960 and the C-terminal domain of Bc0361, respectively. NodB1 is a (βα)_4_β(βα)_3_ barrel (Figure 1 and Figure 3a,b), while NodB2 is a (βα)_8_ barrel (Figure 1 and Figure 3a,c). The catalytic site (ΜΤ1-ΜΤ5), including the metal cofactor binding site and the 2-hydroxyproline position, is spatially identical for both topologies, even though the functionally significant residues or motifs might be provided by different positions in the sequence. 

Topology diagrams were constructed for every NodB domain of our dataset. In Figure 3d, the proteins have been grouped together based on their topological similarities. NodB1 topology incorporates, without exceptions, the proteins belonging to the functional groups of chitin/COS DAs, PG GlcNAc and MurNAc DAs and acetylxylan esterases. In contrast, all those characterized as PNAG DAs and the sole GAC-decorated PG GlcNAc DA follow the NodB2 topology. NodB1 and NodB2 topologies can be further classified into subgroups based on additional, accessory secondary elements occurring within the basic framework of NodB architecture; these confer considerable structural variability to the domain even though the variations cannot be conclusively related to specific substrate recognitions. Some interesting observations comparing evolutionary (Figure 2b) and topological (Figure 3d) classifications are as follows: (i) Topological similarities exist between the turquoise group, the two chitin DAs of insect origin and the more evolutionary distant and uncharacterized 3HFT and 4DWE proteins (Figure 3d). In particular, the fifth strand of the framework structure is completed by an additional pair of strands, consecutive in the sequence, as well. (ii) Likewise, 3WXZ and 4NY2 (Vp and Vc CODs) have an additional pair of strands next to the fifth; this additional pair of strands is provided by the sequence after the fourth strand of the framework structure (Figure 3d). (iii) Evolutionary and topological grouping coincides for the NodB2 proteins 4V33, 4HD5, 6GO1 and 6DQ3.

### 3.3. Sequence Conservation Analysis

**Figure 4:**
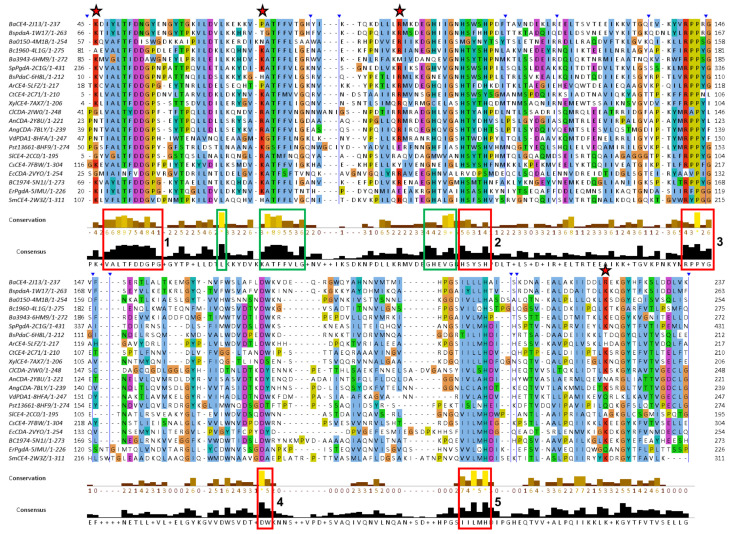
Jalview of T-Coffee multiple sequence alignments for the NodB1.1 members (for a relevant structure, see Figure 1a). Conserved amino acids are highlighted with different colors depending on their physicochemical properties. The vertical blue lines indicate hidden regions. Apart from the ordinary motifs MT1-MT5 (red boxes), this subclass shows additional areas of high conservation, which are indicated by the green boxes. The stars indicate highly conserved basic residues found on the structures’ surfaces.

**Figure 5:**
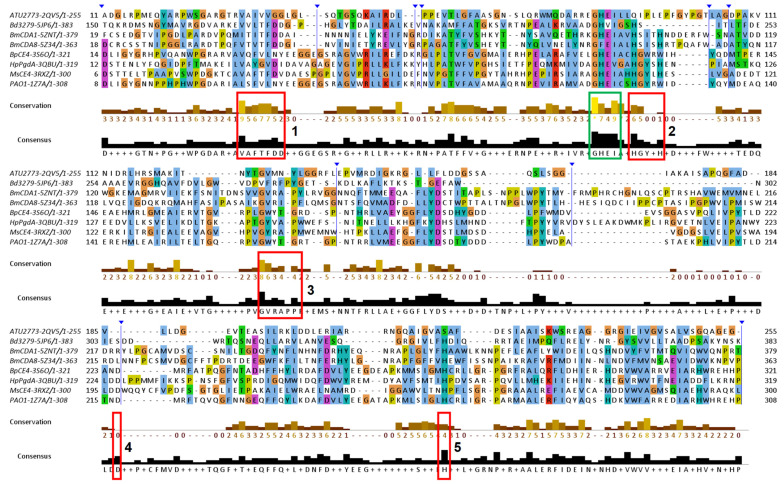
Jalview of T-Coffee/Expresso multiple sequence, structure-aided alignments of the NodB1.2 members. The vertical blue lines and arrows indicate hidden regions of the sequences. The positions of the ordinary motifs MT1-MT5 have been highlighted by the red boxes. Additional areas of noticeable conservation are indicated by the green boxes.

**Figure 6:**
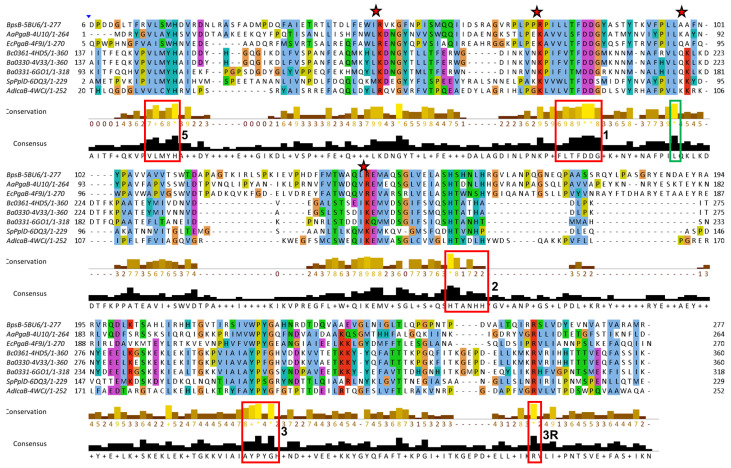
Multiple sequence alignment of the NodB2 members (for a relevant structure, see Figure 1b). The vertical blue lines and arrows indicate hidden regions of the sequences. The positions of the ordinary motifs MT1-MT3 and MT5 have been highlighted by red boxes. Additional areas of noticeable conservation are indicated by green boxes. The stars indicate highly conserved basic residues found on the structures’ surfaces.

Multiple sequence alignments of NodB1 and NodB2 members were performed and presented in Figure 4, Figure 5 and Figure 6. In particular, members of the NodB1 class were aligned in three separate subclasses. The first subclass (hereafter referred to as NodB1.1) includes proteins from the top half of the evolutionary tree (Figure 2b), namely the cyan, red, blue and green groups, as well as the 2CC0, 7FBW and 2VYO proteins (Figure 4). The second subclass (hereafter referred to as NodB1.2) includes proteins from the middle of the evolutionary tree (Figure 2b), namely the turquoise group, as well as the 5JP6, 2QV5, 5Z34 and 5ZNT proteins (Figure 5). The proteins 3HFT, 4DWE, 3WX7 and 4NY2 are classified in a distant subclass of NodB1 topology (NodB1.3, Figure 2b) of high sequence heterogeneity. Members of the distinct NodB2 topology are separately aligned (Figure 6). 

The NodB1.1 subclass members show all the regular features of a CE4 NodB domain, i.e., the five typical motifs MT1-5 (red boxes in Figure 4): MT1 includes two tandem Asp residues serving as catalytic and metal binding sites. MT2 comprises the two histidines, which, in addition to the first D/MT1, bind the catalytic metal. MT3 comprises the highly conserved proline residue, which is converted via a post-translational modification to a 2-hydroxyproline in several CE4 members, thereby playing a key role in the enhancement of the catalytic activity. In addition, a conserved R/MT3 forms a salt bridge/hydrogen-bonding interaction with the catalytic D/MT1. A further, strictly conserved D/MT4 interacts with a conserved H/MT5, forming a catalytically important electron relay. Noticeably, the second proline of MT3 (consensus sequence RPPYG), the aspartic acid of MT4 (DW) and the leucine and histidine residues of MT5 (IILMH) are strictly conserved in all 21 proteins of NodB1.1.

Additional sequence conservations are prominent in NodB1.1, for example, those highlighted by green boxes in Figure 4. A leucine residue/mt6 (first green box; located at α1-helix), the ATFFVLG/mt7 (second green box; located at β2-strand), and GHEVG/mt8 (third green box; loop-β3) are highly conserved. L/mt6, F/mt7 and L and F/MT1 (LTFDDGP) are involved in the formation of a conserved hydrophobic interaction between the secondary structure elements α1, β1 and β2 (outer barrel layer). This interaction is essential for the conformation of the β1α1 loop and, thus, for the correct placement of the metal binding D/MT1, which is located on this loop. EVG residues of mt8 form the third strand (β3) of the barrel, and the preceding, highly conserved GH pair links the second helix (α2) with β3 (α2β3 loop). Thus, the GH sequence places a positive charge at the C-terminus of α2 and provides favorable interaction with its helical moment. This motif comes just before the MT2 loop, which accommodates the two metal-binding histidine residues, and it might be important to ensure its correct conformation and spatial placement. The first F of mt7 and the H/mt8 are involved in a T-packing interaction of their aromatic rings and, together with the L/mt6, participate in the stabilization of a hydrophobic interaction taking place in the space between the barrel and the α-helices.

Unlike NodB1.1, the NodB1.2 subclass gathers proteins of low sequence similarity, which only marginally preserve the NodB sequence features (Figure 5). Thus, the MT1-MT5 motifs can hardly be recognized since they only tend to keep the single active residue from the whole motif sequence. Furthermore, only two (the 3RXZ and 3QBU) of the eight proteins of the group retain all five active residues from each of the five motifs in their sequences. 

**Figure 7:**
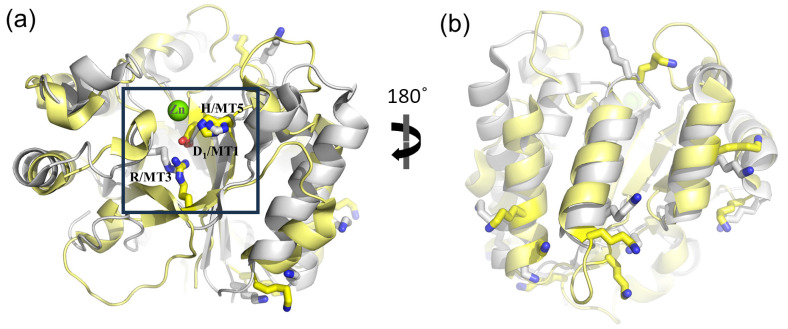
Structural alignment of two representative structures of NodB1.1 (5N1J.pdb, grey color) and NodB2 (C-terminal domain of 4HD5.pdb, yellow color) topologies. The green sphere represents the active site metal. (**a**) Inside the box: the Arg/MT3 residue occupies equivalent positions in the protein’s active site, even though it is provided by different positions in the sequence. (**a**) Outside the box and (**b**): The stick models highlight the position of highly conserved lysine residues between the two proteins, which occupy equivalent positions on the proteins’ surfaces. The structures have been superimposed in PyMOL by the ‘super’ algorithm (RMSD = 2.67 for 110 Ca atoms). (**b**) has been rotated by 180 degrees relative to (**a**).

NodB2 topology keeps most of the MT motifs, with some noticeable differences from the MT motifs of NodB1.1 (Figure 6). MT5 (consensus sequence VLMYH) is the first motif in the sequence, given that it is provided by the amino terminus of the NodB2 fold. MT1 is common between NodB1.1 and NodB2. NodB2/MT2 has the consensus sequence HTX_2_H in the half sample, like the NodB1.1/MT2, and the sequence H(T/S)X_3_H in the rest of the proteins. MT3 (AYPYG) lacks the first proline residue, while the arginine residue is provided from another part of the sequence (Figure 6). However, a structural comparison of a NodB1.1 and a NodB2 protein (Figure 7a) demonstrates that this arginine occupies similar conformation and the same position in the structure relative to the catalytic D/MT1. The MT4 is absent from the NodB2 topology.

Comparing the alignments presented in Figure 4 and Figure 6, it is obvious that NodB1.1 and NodB2 structures share a high similarity of basic residues (Arg and Lys) in specific positions (indicated by stars in both figures), among others. A comparison of two representative structures (Figure 7) shows that these residues are structurally conserved since they line up in the same protein surface, and most of them are located at the very beginning of secondary structure elements.

### 3.4. Function-Related, Structure-Distributed Sequence Conservation 

**Figure 8:**
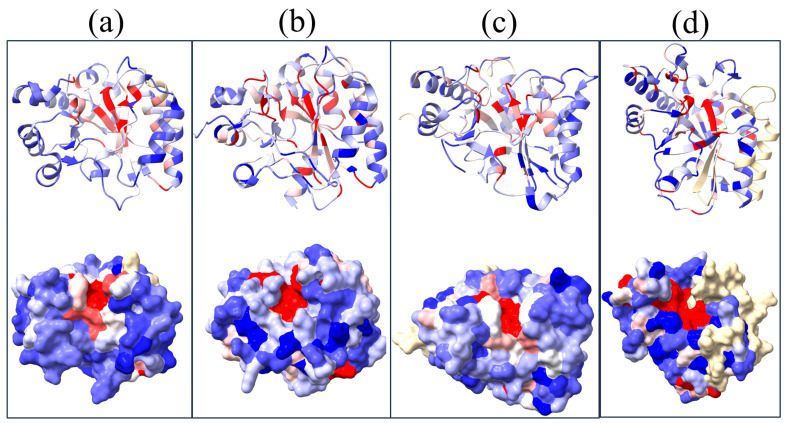
Sequence variability of the NodB domains visualized by the coloring of ribbon diagrams and surfaces of selected CE4 family members. (**a**) GlcNAc PG DA, (**b**) MurNAc PG DA, (**c**) chitin/oligochitin DA and (**d**) PNAG DA family conservations depicted on the representative structures with PDB ids: 4L1G, 1W17, 2IW0 and 4WCJ, respectively (see Section 2). Colors reflect the degree of amino acid sequence conservation calculated by ChimeraX and vary from dark blue (non-conserved) to red (highly conserved). Wheat color represents non-attributed areas.

To validate the observed sequence and structural variabilities of the NodB domain and identify regions that are potentially functionally relevant, a sequence-based 3D cluster analysis was performed within various functionally separate CE4 families. For this analysis, groups of sequences from proteins whose biochemical and enzymatic properties have been sufficiently characterized, such as GlucNAc PG, MurNAc PG, chitin/COS and PNAG deacetylases, were used (Table S2). The sequence conservations into each of the four functional groups have been graphically depicted in four representative structures. In Figure 8, equivalent views of the four structures are presented to facilitate the comparison of the sequence conservation distribution. 

In all four representatives, sequence conservation is not uniformly scattered across the structure. Instead, it is concentrated in specific areas. The catalytic pocket and the substrate binding groove exhibit high conservation within each functional group, dividing the NodB fold into two discrete regions, a conserved one and a variable one (red and blue, respectively, in Figure 8). An inter-grouping comparison shows slight differences in the distribution of the pattern of conservations on the structure. 

As expected, secondary structure elements related to MT1-MT5 display the highest sequence conservation, even though differences are observed in the distribution of the conservation beyond the motifs. In GlucNAc PG deacetylases, the conservation is quite scattered and mainly observed in β strands, such as the full length of β1, β2 and β7 strands, as well as the peptide after β6 (the numbering is according to Figure 1). The loop where the MT3 resides also displays high conservation, as well as the α7β8 loop (Figure 8a). In MurNAc PG deacetylases, the conservation appears even more scattered, extending to or becoming more prominent in short segments (not longer than one turn) of helices (Figure 8b). Chitin/oligochitin deacetylases retain the conservation on β1, β2, β3 and β7 strands, albeit to a lesser extent compared to GluNAc PG DA conservations (Figure 8c). Moreover, this group lacks the extended conservations on loops and helices observed on MurNAc PG DAs. Lastly, PNAGs exhibit limited conservation on β-barrel’s residues, with the most significant conservation found on the β1 strand. Conversely, helical parts of the structure, such as the MT3 located turn, display the most prominent conservation within the fold. 

### 3.5. NodB Fold beyond the CE4 Family

Proteins of the NodB1.2 subclass not only have relatively low sequence similarities and low conservation of the typical NodB sequence motifs (MT1-MT5), but their exact biological function awaits experimental confirmation (Figure 5). For instance, most of the NodB1.2 members fall into the group of proteins with ‘non-experimentally identified substrate’ in Table S1. 

#### 3.5.1. NodB in PuuE Allantoinases

**Figure 9:**
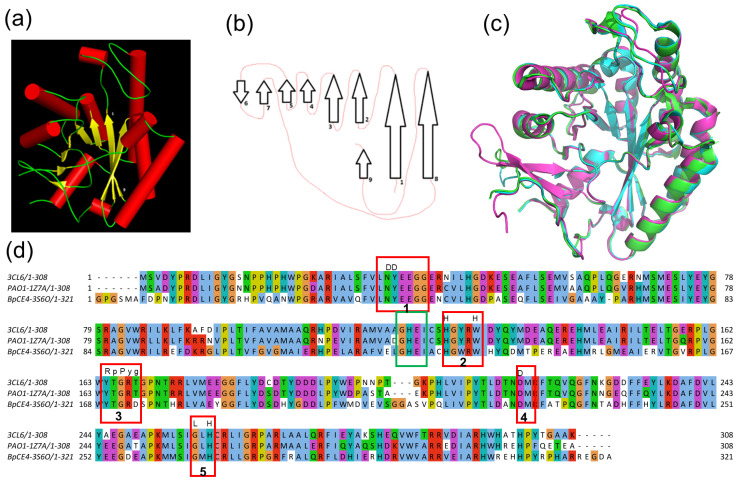
PuuE-like allantoinase 3CL6 in comparison to two close relatives of the NodB1.2 subclass. (**a**) The 3D structure of 3CL6 in cartoon representation (the coloring is according to secondary structure, i.e., helices are red cylinders and strands are yellow arrows). (**b**) A simplified (without the helices) topology diagram of 3CL6. (**c**) Structural superposition of 3CL6 (green), 1Z7A (cyan) and 3S6O (magenta). (**d**) Jalview of TCOFFEE multiple sequence alignment for the NodB domains of the three proteins. Conserved amino acids are highlighted with different colors depending on their physicochemical properties. The red boxes highlight the position of the classical MT1-MT5 motifs. The residues on top of the sequences highlight the conserved residues in these motifs in NodB1.1 for comparison. MT1, 2 and 3 display great differences. MT1 lacks both aspartic acid residues, and MT2 abolishes the second histidine residue, which has been replaced by a tryptophane. These changes are consistent with the fact that 3CL6 is a non-metal-dependent enzyme. MT3 lacks the highly conserved proline residue, which has been shown to be able to be post-translationally hydroxylated for active site maturation. In contrast, MT4 and MT5 retain the catalytically significant aspartic acid and histidine residues, respectively.

Interestingly, two proteins in our NodB1.2 subclass, i.e., 1Z7A (*Pseudomonas aeruginosa* PAO1) and 3S6O (*Burkholderia pseudomallei* BpCE4), have important sequence and structural similarities with an alternative, PuuE allantoinase (PDB id 3CL6; Figure 9a). Indeed, the similarities of this non-metal-dependent, alternative allantoinase with CE4 NodB proteins have been previously reported since its structure was determined by molecular replacement (X-ray crystallography) using the structure of 1Z7A as the search model [22]. The topological diagram of 3CL6 (Figure 9b) is similar to those of 1Z7A and 3S6O, with a β-hairpin insert after the β5 strand being the main structural variation compared to the classical topology. The multiple sequence alignment and structural superposition of the three proteins confirm a significant structural similarity (Figure 9c,d). 3CL6 is superimposed to 1Z7A and 3S6O with an rmsd of 0.3 for 262 Ca atoms and 0.5 for 243 Ca atoms, respectively. Such a high degree of similarity raises questions as to whether 1Z7A and 3S6O are indeed CE4 members or, instead, PuuE-like allantoinases of *P. aeruginosa* and *B. pseudomallei,* respectively.

Furthermore, it should be noted that a DALI search of the PDB using the 3CL6 structure as a query identifies 5Z34 (insect chitin deacetylase) as a very close structural relative.

#### 3.5.2. NodB in the Wider Family of Carbohydrate Esterases

**Figure 10:**
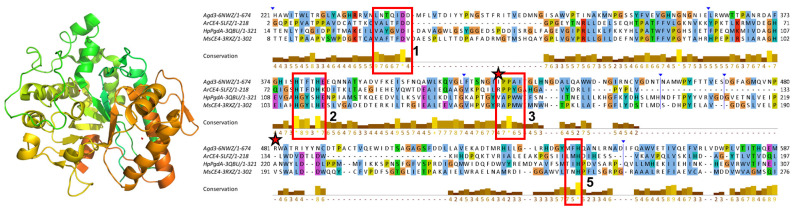
The CE18 founding member Agd3 (6NWZ) protein. Left, a cartoon representation of the structure. The coloring scheme is from N- (green) to C-terminus (brown). Right, a multiple sequence alignment (structure-aided) with three relatives from the CE4 NodB1 domain (Jalview/TCOFFEE). Conserved amino acids are highlighted with different colors depending on their physicochemical properties. The red boxes highlight the position of the classical MT1-MT5 motifs, which show significant variations. MT1 retains the catalytic aspartic acid. MT2 retains both histidines. MT3 retains the proline pair, but the catalytically significant arginine is given by another position of the sequence (marked by the stars), as it was determined by a structural alignment of Agd3 with PNAG CE4 deacetylases and the fact that this arginine coordinates the putative catalytic base/MT1 [21]. MT4 is absent, while MT5 retains the catalytically significant histidine residue.

In the last few years, several proteins containing NodB-like domains have emerged and been reported either as founding members of new CE families or as non-classified carbohydrate esterases. *Aspergillus fumigatus* Agd3 (PDB id 6NWZ) is a metal-dependent, exopolysaccharide deacetylase required for biofilm formation and virulence [21]. The Agd3 catalytic domain folds as a NodB-like domain, even though it has a low sequence similarity with the classical CE4/NodB1 and lacks the canonical MT4 (Figure 10). Agd3 has only a distant homology to the functional CE4 homologs, namely, the PNAG proteins (NodB2), which are involved in biofilm formation in bacteria (Table S1). The best sequence match from the CE4 family is the NodB1.1 ArCE4 (5LFZ) protein, and the closest structural relatives are the NodB1.2 3QBU and 3RXZ proteins (Figure 10). Agd3 is the founding member of the new carbohydrate esterase family CE18 [21].

*Klebsiella pneumoniae* ChbG is a chito-oligosaccharide deacetylase that catalyzes the removal of one acetyl group from *N*,*N*’-diacetylchitobiose [40]. The protein has no sequence similarity with other chito-oligosaccharide deacetylases; therefore, it is characterized as a non-classified carbohydrate esterase. A structural search identifies the NodB1.1 2C1G and 4NY2 as the closest CE4 family relatives. However, as far as the existence and identity of canonical motifs (MT1-MT5) is concerned, many variations are observed [40].

ArnD protein is involved in the biosynthesis of 4-deoxy-4-amino-L-arabinose (Ara4N). Ara4N covalently modifies lipid A, making the bacteria resistant to antimicrobial peptides and polymyxin antibiotics [41]. The recent structure determination of *Salmonella typhimurium* ArnD (StArnD) revealed a NodB1-like domain. However, low sequence similarity and several distinct structural features led to the classification of this protein in a new family of membrane-associated carbohydrate esterases. A structure-based search of the database shows that the NodB1.2 3RXZ, 3QBU, 1Z7A and 3S6O are the closest relatives (rmsd~2.4), even though the sequence identity is 19% or less.

## 4. Discussion

The classical NodB domain can adopt one of two possible topologies, namely NodB1 and NodB2, representing evolutionarily and structurally discrete classes. Clustering analysis highlights certain proteins such as 3HFT, 4DWE, 3WX7 and 4NY2 as significantly diverging NodB1-like folds. Likewise, correspondence analysis of structural comparisons (Figure S1) clearly distinguishes the two topologies (NodB1 and NodB2) and at least the NodB1.1 subclass as structurally distinct entities along the first and second eigenvector, respectively. In the dataset that we analyzed, all CE4 PNAG deacetylases belong to the NodB2 topology without exceptions. Although this observation suggests common or related activities among all NodB2-type enzymes, it cannot be verified yet, as key enzymatic data are missing. 

NodB1.1 encompasses all the other CE4 functionalities and incorporates all the standard characteristics of the NodB domain as described in the literature, while NodB1.2 is a quite heterogeneous—in sequence and structure—subclass that consists of proteins of uncharacterized yet functions. Among other characteristics, members of NodB1.2 abolish the Ser/Thr residue, which usually precedes the second H/MT2 in NodB1.1, and motifs MT4 and MT5 retain only the catalytically significant residues Asp and His. Key to the identification of the MT3 motif is the residue occupying an equivalent position to the 2-hydroxyproline (2-Hyp) residue systematically present in NodB1.1. Structural superpositions reveal that in NodB1.2, this position is a Pro or Gly; interestingly, the preceding residue adopts a backbone conformation such that the carbonyl oxygen points to the active site, suggesting its potential involvement in the catalytic reaction, akin to the -OH group of 2-Hyp.

The similarities observed in motifs MT4 and MT5 between the catalytically inert Ba0150-4M1B and the MurNAc peptidoglycan deacetylases BsPdaA-1W17/BaCE4-2J13 might support that the protein has evolved from a MurNAc rather than a GlucNAc PG deacetylase ancestor. The EcCDA-2VYO lacks both histidines of the metal co-factor site (MT2) and the catalytic Asp of MT1, as well as the Arg of MT3; thus, it is probably an inactive polysaccharide deacetylase.

NodB2 proteins retain some of the main features of the conserved motifs. In contrast to NodB1, MT5 is located at the N-terminus, although it retains the same spatial position. The active site NodB1 R/MT3 is replaced by another Arg, which protrudes to the active site from a different position (see Figure 6). In both cases, the Arg residue interacts with the catalytic D/MT1, stabilizing it in the same position and orientation in the active site. MT4 is not clearly defined in NodB2; its key feature, an Asp interacting with the conserved H/MT5, is also missing, with the exception of BpsB-5BU6.

Upon visual inspection, it is apparent that in AaPgaB-4U1O, the His residue from MT5 participates in a second metal binding site, along with the catalytic Asp from MT1 and a conserved water molecule. Putative second metal binding sites are also present in EcPgaB-4F9J and IcaB-4WCJ, involving residues from MT1 and MT5. 

## 5. Conclusions

CE4 NodB architecture adopts two distinct topologies, NodB1 and NodB2. NodB2 is less populated and has been exclusively related to CE4 de-*N*-acetylases of poly-β-1,6-*Ν*-acetyl-*D*-glucosamine. Therefore, we propose that the enzymatically uncharacterized members of the group may exert analogous functions. Within the NodB1 topology, the framework structure bears variations that are clearly related to substrate specificity and functions. A careful examination of the active site formation highlights additional conserved motifs to the classical MT1-MT5, suggesting versatility to the substrate recognition, binding and catalysis. In particular, NodB1.2 is a diverged, heterogeneous subclass that includes proteins with unknown (so far) specific substrates, although, for some of them, common CE4 substrates have been unsuccessfully tested. We speculate that these proteins are active in different sets of substrates, other than the classical carbohydrates. Indeed, progressively increasing evidence supports this hypothesis. In recent years, at least three proteins that fold in NodBs have been studied, and none of them belong to the CE4 family. Two of them are carbohydrate esterases (CE18 family and membrane-linked carbohydrate esterase); while, the third has been reported as an atypical PuuE-like allantoinase processing a quite different substrate.

## Data Availability

All structures used are publicly deposited and readily available from the PDB database.

## Supplementary Materials

The following supporting information can be downloaded at: https://www.mdpi.com/article/10.3390/biom14030325/s1, Table S1. NodB-domain containing CE4 proteins extracted from the Protein Data Bank before July 2023. Our analysis was performed at the non-redundant NodB-domains of the underlined structures. PG, DA and MD stand for PeptidoGlycan, DeAcetylase and Molecular Dynamic simulations, respectively; Table S2. CE4 protein sequences with adequately characterized functions. These proteins were used for generating sequence-based, function-related conservation clustering. The information has been depicted in the 3D structures of representative members (see Figure 4); Figure S1. Corresponding analysis classification (all against all analysis through DALI server) of the protein sample presented in Table S1.
