# Supplementary material for: Variations of the NodB Architecture Are Attuned to Functional Specificities into and beyond the Carbohydrate Esterase Family 4
Source: Biomolecules. 2024 Mar 8;14(3):325. doi: 10.3390/biom14030325 (PMC10967932; doi:10.3390/biom14030325)
Supplement: Supplementary file 1 [file biomolecules-14-00325-s001.zip › Supplementary.pdf]

SUPPLEMENTARY INFORMATION

Variations of the NodB architecture are attuned to functional specificities into and beyond the CE4 family

Alexis S. Molfetas, Boutris Nikiforos, AnastasiaTomatsidou, Michael Kokkinidis & Vasiliki E. Fadouloglou

Table S1. NodB-domain containing CE4 proteins extracted from the Protein Data Bank before July 2023. Our analysis was performed at the non-redundant NodB-domains of the underlined structures. PG, DA and MD stand for PeptidoGlycan, DeAcetylase and Molecular Dynamic simulations, respectively.

**Subgroups of CE4 family**	**Protein name/family**	**PDB code**	**Organism**	**Activity data**
**Chitin/Oligochitin DA**	ClCDA	2IW0	*Colletotrichum lindemuthianum*	Active on (GlcNAc)$_{3-6}$ and partially deacetylated chitooligosaccharides
	AnCDA	2Y8U	*Aspergillus nidulans*	Active on (GlcNAc)$_{2-6}$, chitosan, crystalline chitin, acetylxylan.<br>Inactive on GlcNAc, peptidoglycan
	VpCOD	3WX7	*Vibrio parahaemolyticus*	Active on (GlcNAc)$_{2-4}$
	VcCOD	4NY2, 4NYU, 4NYY, 4NZ1, 4NZ3, 4NZ4, 4NZ5, 4OUI[	*Vibrio cholerae*	Active on (GlcNAc)$_{2}$
	ArCE4	5LFZ, 5LGC	*Arthrobacter species*	Active on (GlcNAc)$_{3-6}$, chitosan, α- and β-chitin, acetylxylan. Low activity on (GlcNAc)$_{2}$
	BmCDA8	5Z34	*Bombyx mori*	Active on (GlcNAc)$_{3-6}$
	BmCDA1	5ZNT, 5ZNS	*Bombyx mori*	Deacetylates ethylene glycol chitin in the presence of accessory proteins
	AngCDA	7BLY	*Aspergillus niger*	Active on GlcNac$_{2-6}$, weak activity on α-chitin and β-chitin, slight increase on colloidal chitin
	Pst_13661	8HF9,8HE1, 8HE2,8HE4	*Puccinia striiformis*	Gene knock-out makes it inactive against chitin.

	VdPDA1	8HFA	*Puccinia striiformis*	Gene knock-out makes it inactive against chitin
**PG GlcNAc DA**	SpPgdA	2C1G, 2C1I	*Streptococcus pneumoniae*	Active on peptidoglycan
	Bc1960	4L1G, 5O6Y	*Bacillus Cereus*	Active on peptidoglycan (from gram$^{+/-}$ bacteria), N-acetyl-D-glucosaminyl-(β-1,4)-N-acetylmuramyl-L-alanyl-D-isoglutamine, $(GlcNAc)_{4\text{-}6}$, glycolchitin
	Bd3279	5JP6	*Bdellovibrio bacteriovorus*	Active on peptidoglycan
	Bc1974	5N1J, 5N1P, 5NC6,5NC, 5NCD, 5NEK, 5NEL	*Bacillus Cereus*	Active on $(GlcNAc)_5$
**PG MurNAc DA**	BsPdaA	1W17, 1W1A, 1W1B, 1NY1	*Bacillus subtilis*	Active on peptidoglycan. Protein/NAG complex (crystallography).
	BaCE4	2J13	*Bacillus Anthracis*	Putative activity. No biochemical characterization is provided; however, the protein is considered to be the *B. anthracis* ortholog of BsPdaA.
**PG MurNAc & oligochitin DA**	BsPdaC	6H8L, 6H8N	*Bacillus subtilis*	Active on $(GlcNAc)_{4\text{-}5}$ & chitooligosaccharide kinetics
**Acetylxylan esterases**	SlCE4 (SlAxe)	2CC0	*Streptomyces lividans*	Active on 2-O-acetyl-4-nitrophenyl -D-xylopyranoside
	CtCE4 (CtAxe)	2C71, 2C79	*Clostridium thermocellum*	Active on2-O-acetyl-4-nitrophenyl -D-xylopyranoside
	XylCE4	7AX7	*Trinervitermes trinervoides*	Active on p-NPA (4-nitrophenyl acetate)
**Poly-ß-1,6-GlcNAc DA**	AdIcaB	4WCJ	*Ammonifex degensii*	Active on $(\beta\text{-}1,6\text{-}GlcNAc)_4$ and $(\beta\text{-}1,6\text{-}GlcNAc)_5$
	EcPgaB	3VUS, 4F9J, 4F9D, 4P7L, 4P7N, 4P7O, 4P7Q, 4P7R	*Escherichia coli*	Active on β-1,6-GlcNAc oligomers
	AaPgaB	4U10	*Aggregatibacter actinomycetemcomitan*	Active on 7-acetoxycoumarin-3-carboxylic acid
	BpsB	5BU6	*Bordetella bronchiseptica*periplasmic	Active on $\beta\text{-}1,6\text{-}(GlcNAc)_{1\text{-}5}$
**PG GAC linkage DA**	SpPplD	6DQ3	*Streptococcus pyogenes*	de-N-acetylase of the cell wall linkage of streptococcal rhamnopolysaccharides
	SmCE4	2W3Z	*Streptococcus mutans*	Active on $(GlcNAc)_{6.}$ No activity was observed toward shorter chitooligosaccharides or a synthetic peptidoglycan tetrasaccharide. Authors suggest activity on as-yet unidentified polysaccharide.
	Bc0361	4HD5	*Bacillus Cereus*	-
	Ba0330	4V33	*Bacillus Anthracis*	Inactive on glycol chitin, $GlcNAc_{2\text{-}6}$, p-nitrophenyl acetate, N-acetyl-D-

				glucosaminyl-(β-1,4)-N-acetylmuramyl-L-alanyl-D-isoglutamine
Substrate identification awaits experimental confirmation	Ba0331	6GO1	*Bacillus Anthracis*	-
	PAO1	1Z7A	*Pseudomonas aeruginosa*	-
	HpPgdA	3QBU, 4LY4	*Helicobacter pylori*	Inactive on GlcNAc, $GlcNAc_3$, N-acetyl putrescine, N-acetyl spermidine, N-acetyl cadaverine, N-acetyl dipeptides, allantoin
	Bb0128	3HFT	*Bordetella bronchiseptica*	-
	MsCE4	3RXZ	*Mycobacterium Smegmatis*	-
	BpCE4	3S6O	*Burkholderia pseudomallei*	-
	Bacova_03992	4DWE	*Bacteroides ovatus*	-
	EcCDA	2VYO[**Error! Reference source not found.**]	*Encephalitozoon cuniculi*	Inactive on chitooligosaccharides and crystalline β-chitin. EcCDA exhibited no de-N-acetylase activity on neither of the substrates tested
	ErPgdA	5JMU	*Eubacterium rectale*	-
	CsCE4	7FBW	*Caldanaerobacter subterraneus*	-
	ATU2773	2QV5	*Agrobacterium tumefaciens*	-
**Pseudo deacetylases**	Ba0150	4M1B	*Bacillus Anthracis*	No activity
	Ba3943	6HM9, 6HPA 7BKF	*Bacillus Anthracis*	-

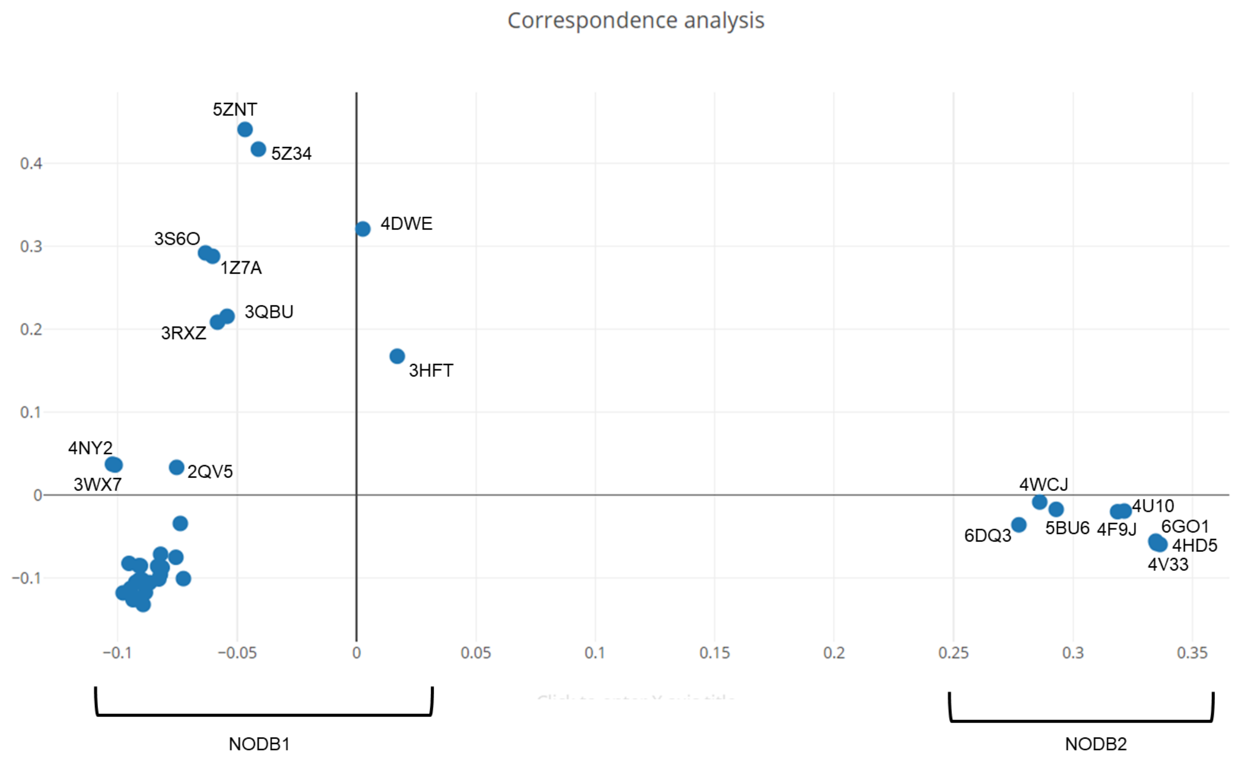

Figure S1. Corresponding analysis classification (all against all analysis through DALI server) of the protein sample presented in Table S1.

Table S2. CE4 protein sequences with adequately characterized functions. These proteins were used for generating sequence-based, function-related conservation clustering. The information has been depicted in the 3D structures of representative members (see).

GlcNAc PG deacetylases:

>Bc1960-4L1G
MFAPYQWGLERDVSYAYMPYNSFYYGDYINSLPYAYIPQNYEVQMKADDRGSWTPFSWVEKYAYAFSGPYNKAEVALTFDDGPDLE
FTPKILDKLKQHNVKATFFLLGENAEKFPNIVKRIANEGHVIGNHTYSHPNLAKVNEDEYRNQIIKTEEILNRLAGYAPKFIRPXYGEILE
NQLKWATEQNFMIVQWSVDTVDWKGVSADTITNNVLGNSFPGSVILQHSTPGGHLQGSVDALDKIIPQLKTKGARFVTLPSMFQTSKE
RK
>BC1974
MEKALKIKQIVVVLIAIAAVAIGYYMFQSITSPAKAVAKQENVVQLASEQPKVEMNKTAPSRFNGKERKVAYLTFDDGPGKYTAELLTL
KQHDAKATFFLIGANVKEFPDLVKRENAEGHYVGMHSMTHNFAKLYKNGEYVNEMKEDQGLIANIIGKSPKLTRPPYGSMPGLNEGN
KVVEGGFKVWDWTIDSLDWRYNKMPVDAAAAQIAQNVLTNATKPQEVILMHDIHPQSVAAVPAILKGLKEKGYEFEAYHEESHFPVN
FWHDNRM
>BC3618
MLLRKELEPTGYVTWEVPNNEKIIAITFDDGPDPTYTPQVLDLLRQYKAEATFFMIGFRVQRNPYLVKQVLKEGHEIGNHTMNHLYASN
SSDEKLENDILDGKKFFEKWVKEPLLFRPPGGYINDAVFKTAKEAGYQTVLWSWHQDPRDWANPGVESIVNHVVKNAKSGDIVLLHD
GGNDRSQTVAALAKILPELKKQGYRFVTVSELLRYKH
>BC2929

MHNGIRMTTLVKRAMLVCAGVLFTYEVAFGSVHTALASTEDEAKSVQLVSEIQTSLAPKEAPKHYNGQVRKVAYLTFDDGPGKYTAE
LLDMLKKENAKATFFLIGSNVKAFPDLVKREDAEGHYVGMHSMTHNYKKLYTEGHYVDEMKEDQGLIAGVLGKSPVLTRPSYGSMP
GLNEALRNKVVENGLKVWDWTIDSLDWKYNKMQVDAASAKIVENVLHGATNPTEVILMHDIHPQSVKAVPGIIKGLKEKGYELEAYN
ENEHFPLNFWHDNRM
>BC5204
MIKRVFQCIILFLTITMYVSSNTEATTVIPAEYHPNTETTSPTQKIAYLTFDDGPNKYTTQILNILKEKNGKATFFVIGGKVPHYKKTMQRL
IKDGHYIGLHSMSHDVKRLYTGDPSTLIAEMEQTQSIVQQVTKLNTHLVRVPYGSMPYLKKNYRDALVSAQYKMWDWTIDTYDWKS
YDNPSAILERVRNQSDEQVEVILMHDSSVTVQILPKVIDYLQSQGYKLLPYNPSSHLKVNFWKDTRL
>BsPdaC
MLAKRIKWFHVLIAVVCVVGLIGFFHNHSLKKETVMNKVRTDSQYGNVEIATLVNDGKTFNYAVNYPVFKNEKMDSALKRFAEKEVR
QFQKETKDVDQEHTTKRNELNVDYKIVHYAKQTVAIVFNEYKYIGGAHGQTVKKTFNYDFSKQAFLSIDDIFKEDADYLHKLSLIAYHE
LKKNKDIAADDALLKEGTAPKKENFSRFAIKEDYIELYFDTYQVAAGYLGEQSIAIKKSLLKDILKEQYIDKAKNKNKIKEQKPKHEVIS
LPKEETVDPNQKVIALTFDDGPNPATTNQILDSLKKYKGHATFFVLGSRVQYYPETLIRMLKEGNEVGNHSWSHPLLTRLSVKEALKQI
NDTQDIIEKISGYRPTLVRPPYGGINDELRSQMKMDVALWDVDPEDWKDRNKKTIVDRVMNQAGDGRTILIHDIYRTSADAADEIIKKL
TDQGYQLVTVSQLEEVKKQREAK
>HP0310
MAKEILVAYGVDIDAVAGWLGSYGGEDSPDDISRGLFAGEVGIPRLLKLFKKYHLPATWFSPGHSIETFSEQMKMIVDAGHEVGAHGY
SHENPIAMTAKQEEDVLLKSVELIKDLTGKAPTGYVAPWWEFSNITNELLLKHGFKYDHSLMHNDFTPYYVRVGDSWSKIDYSLEAKD
WMKPLIRGVETDLVEIPANWYLDDLPPMMFIKKSPNSFGFVSPHDIGQMWIDQFDWVYREMDYAVFSMTIHPDVSARPQVLLMHEKII
EHINKHEGVRWVTFNEIADDFLKRNPRKK
>CdPdaV
MRNKKKKINRKKLYLLLSAIVVCLAFIVVGVRVSSLNMKENEISRNAKLSSSTITDIVSNTSIEVSKGPSKSSGKIAYITIDDGPSKFTDQMI
KTLNKYNVKATFFMIDGNMKEYPQQVKNIIKNGNTAGFHSVSHDIHKLYVTSTSAKEEFDTNDQTFYKITGKHSKVIRIPYGSKPYTPQ
ASYQALVDAGYKIWDWDLDTEDWRSNSSQIVQNVKNHIKNRKGEDKDQLVVLMHEKKQSAEALDSVLKFLSDEGYEFAPVDQNQIP
KNYWLRNLE
>SpPgdA
MNKSRLGRGRHGKTRHVLLALIGILAISICLLGGFIAFKIYQQKSFEQKIESLKKEKDDQLSEGNQKEHFRQGQAEVIAYYPLQGEKVISS
VRELINQDVKDKLESKDNLVFYYTEQEESGLKGVVNRNVTKQIYDLVAFKIEETEKTSLGKVHLTEDGQPFTLDQLFSDASKAKEQLIK
ELTSFIEDKKIEQDQSEQIVKNFSDQDLSAWNFDYKDSQIILYPSPVVENLEEIALPVSAFFDVIQSSYLLEKDAALYQSYFDKKHQKVVA
LTFDDGPNPATTPQVLETLAKYDIKATFFVLGKNVSGNEDLVKRIKSEGHVVGNHSWSHPILSQLSLDEAKKQITDTEDVLTKVLGSSSK
LMRPPYGAITDDIRNSLDLSFIMWDVDSLDWKSKNEASILTEIQHQVANGSIVLMHDIHSPTVNALPRVIEYLKNQGYTFVTIPEMLNTR
LKAHELYYSRDE
>SsPgdA
MKKVSFWLGINIALLGIMVSLAVWLFAGLQERQVSQFIEEKQQTILAKGKGKIQEGNIDTTHVVAALPTDDAGHVLGPVESRMISYVQR
RFGHKKPAGKIQKLVFVSSIEGKTNFKNVTAREIQAEHYKVDNLQIKKQDKLPSERVLLTQDNELFTLEDLLPNLSSAASIIVDHLREALL
AQGMKETDVEAIVKKFETLDLNAISFSYGDSQLTLQLPDGYGINQLVLPISDLYPVVKSDYLVDADKVGYDEYMAAQVVDKKHLRQV
ALTFDDGPNPNTTPVVLDLLKKYNAKATFFVVGKAVVGNEAILRRMVAEGHVIANHTWNHPNLVTISGEQVQREIQDTQAAITEATGI
VPTMVRPPYGSVNQAVVNQMGLPSIYWSVNSKDWKSRNPQAILKEIKEQTCPGSIILMHDIHQSTVDSLESVLQYLTGEGYNMVTVTD
LLASPLNPQLIYYSQELSGPAQ
>LmPgdA
MKIRWIRLSLVAILIIAVVFIGVIGFQKYQFSKSRNKVIMQMDRLMKDQDGGNFRRLDKKENGVEIISYIPKTTEKKDNEIIQKEIGKATD
AEVKKLNRDKETQGIIFYTYQKHRMAEQAISYKAVQSEYVKEGRTKFVLKDKKDICKNIVTDAETGALLTLGEVLIKSNQTKLNLKTAV
EEELIKTGDFSLKDVGNLGKIKSLVKWNQTDFEITNSEIILPVKIPGAPEPKKVKVKLADIASSVNKRYLPSSVKVPEVPKAKTNKRIALTF
DDGPSSSVTPGVLDTLKRHNVKATFFVLGSSVIQNPGLVKRELEEGHQVGSHSWDHPQLTKQSTQEVYNQILKTQKAVFDQTGYFPTT
MRPPYGAVNKQVAEEIGLPIIQWSVDTEDWKYRNAGIVTKKVLAGATDGAIVLMHDIHKTTAASLDTTLTKLKSQGYEFVTIDELYGE
KLQIGKQYFDKTDSRMVK
>LlPgdA
MKQRRLKSKGAKKAFWKLTLLIGALLIVVAGVGTYYYDQGVQKQKIAYEKQVDEARNAKDKAYDSRKANDIEQAHKLIDKLHVKDK
TSLDKSMSQLTNYLSEIDKVNQIVAKAHENISEEGIKSSEEALELLKAPYEKSDKEALAKQVNADKQILIERQKEAELIANVQNQYANKK
LIALTFDDGPNPNTTPQLLKIFSDAQVPATFFALGKQAQACPQIIKEEADRGNEVASHTWDHKDLVTLSPDQQKQEIESANQLINKITGN
VTLFRPPYGSYNKSVLSQTNLSAVNWTVDTNDWRYRTSAPVVQNVSTYAHDGAIILMHDIHQWSVDAVPQIIKNLKAQGYTFVTVSTL
LTVQDGGAKAQQVYFGR
>EfPgdA
MRHKKRKFKINGYVLFSALILIVLVFIQTFLLTQPKITSKNDDTTQTSTKNSTAKKDSPTKLEKESTHMTISSKKYAYSADKIRKYITGEEP
YKGEKLVFLTFDDGVNNQITPQVLDTLKKYNVHATFFLVGNTLTSENQKIVKREVAEGHSIGFHSSTHDYATLYPNGIVNTKEIQAEIAT
MENSLKKILGETFQTTLWRYPGGHMSWGGTEKSDELFKQLGIHWIDWNAMVGDAEPLDRQPTTVAEMLAFHQHSLEVYPDYNIRVVL
MHDSVDKELTKQALPQLIEFYQANGYQFGVLY
>Bd0468
MMKRPISLALSALTLTASLAHAQEEALPQAPSNEKLIELQTTQGMKRAMEKILNIESEWGIFDKFKPNRCKYAIQVPDSVTRGTIALTFD
DGPNPDTTPLILDVLKAHNAKGTFFILGSKIKGNEAILRRMVAEGHQLANHSYSHPNFHELSSSRMNSEIKTTDRLLRTVGTPRFFRYPY
GNSTCSSNELVSSLGYVNVGWDIDTCDWAFADGQVSDKENATCQAPQHLRRDYAGYVANVVAQTQGGVLLMHDIHKNTAHSLDRL
MTMLEQDGYRFVSLTDRNIFPKLNRR
>Bd3279
MYMKKLVFGGMLIVSAASLVGCGSQIGSSVRQAVSDNQSAQTLVEWENSEANPEALFANWRHEFMVDSSKRESMKTELCKELQALPQ
DLTLFENEIRDENNRALVSGCKEELLAQVDEHFDEQRESMSVPGHALKAVQSRNSFRFPDNTQKRDMSNGYMAVRGDVARKEVVLTF
DDGPHGLYTDAILRALKEVNAKAMFFATGKSVRTNPEALKRVAADGHVIGSHSITHACLGTSVACYKQMGNRNLTFDEAAAEVRGGQ
AVFDVLGWVDPVFRFPYGETSKDLKAFLKTKSTGEFAWNIESDDWRTQSNEQLLARVLANVESQGRGIVLFHDIQRRTAEIMPQFLREL
YNRGYSVVLLTAADPSAKYNSKLVKRKQQLP

MurNAc PG deacetylases:

>BsPda-1W17
MKWMCSICCAAVLLAGGAAQAEAVPNEPINWGFKRSVNHQPPDAGKQLNSLIEKYDAFYLGNTKEKTIYLTFDNGYENGYTPKVLDV
LKKHRVTGTFFVTGHFVKDQPQLIKRMSDEGHIIGNHSFHHPDLTTKTADQIQDELDSVNEEVYKITGKQDNLYLRPPRGVFSEYVLKET
KRLGYQTVFWSVAFVDWKINNQKGKKYAYDHMIKQAHPGAIYLLHTVSRDNAEALDDAITDLKKQGYTFKSIDDLMFEKEMRLPSL
>BsPdaC
MLAKRIKWFHVLIAVVCVVGLIGFFHNHSLKKETVMNKVRTDSQYGNVEIATLVNDGKTFNYAVNYPVFKNEKMDSALKRFAEKEVR
QFQKETKDVDQEHTTKRNELNVDYKIVHYAKQTVAIVFNEYKYIGGAHGQTVKKTFNYDFSKQAFLSIDDIFKEDADYLHKLSLIAYHE
LKKNKDIAADDALLKEGTAPKKENFSRFAIKEDYIELYFDTYQVAAGYLGEQSIAIKKSLLKDILKEQYIDKAKNKNKIKEQKPKHEVIS
LPKEETVDPNQKVIALTFDDGPNPATTNQILDSLKKYKGHATFFVLGSRVQYYPETLIRMLKEGNEVGNHSWSHPLLTRLSVKEALKQI
NDTQDIIEKISGYRPTLVRPPYGGINDELRSQMKMDVALWDVDPEDWKDRNKKTIVDRVMNQAGDGRTILIHDIYRTSADAADEIIKKT
DQGYQLVTVSQLEEVKKQREAK
>ClPdaA1
MKKDSLKKYIMIGAFALILFGIASINFKSLDKTKTQISSPTLDTHEYDWYFNPREDGKQPSPIKEADFFKKYGAYYVGNPNEKVIYLSFDA
GYESGNTPKLLDTLKKHNAKAQFFVVESYIKSNPELIKRMEKEGHLVCNHSKSHPSMAGITDFEKFKEEITSVEKAYKDVTGKEMPKYF
RPPMGKFSEQSLKYTQDLGYKSIFWSFAYVDWYEKKQPTHEFAKNKIYSRTHPGAIVLLHPNSSTNTEILDEVLTHWEKEGYKLKTLDY
LNNKK
>ClPdaA2
MFRKVLHYLTLLSISIFFIVGCSNSQNNQNENQNKETQLQEDKEKIDSGKDTSNVIVSDGTDKPSKATTNNDNNKLDVSSLDNTTLDWF
YIPNNKHKTPEVNTDIEFKFSDYDALYNGPTKDGQKTLYLTFDEGYENGYTTKILDTLKQNQVKAVFFVTAPYIKENKDLVKRMVSEG
HIVGNHSKTHPSMPTKTSNLKNFNDELYDVEKLYKDVTGKDMVKFFRPPMGKYSEKSLAMTKNLGYKTVFWSFAYRDWDTDKQPSH
EEATQKIMDNLHDGSILLLHAVSKTSTEILNDFISNARKLGYEFELLEY
>BaCE4-2J13
MAYTNTPHNWGIPRPKNETVPDAGKLYTDLLQKNGGFYLGDTKKKDIYLTFDNGYENGYTGKILDVLKEKKVPATFFVTGHYIKTQDL
LLRMKDEGHIIGNHSWSHPDFTAVNDEKLREELTSVTEEIKKVTGQKEVKYVRPPRGVFSERTLALTKEMGYYNVFWSLAFLDWKVDE
QRGWQYAHNNVMTMIHPGSILLLHAISKDNAEALAKIIDDLREKGYHFKSLDDLVKSNQP

Chitin/oligochitin deacetylases:
>ClCDA-2IW0
MHFSTLFGAAATAALAGSTNASPLARRQVPVGTPILQCTQPGLVALTYDDGPFTFTPQLLDILKQNDVRATFFVNGNNWANIEAGSNPD
TIRRMRADGHLVGSHTYAHPDLNTLSSADRISQMRQLEEATRRIDGFAPKYMRAPYLSCDAGCQGDLGGLGYHIIDTNLDTKDYENNK
PETTHLSAEKFNNELSADVGANSYIVLSHDVHEQTVVSLTQKLIDTLKSKGYRAVTVGECLGDAPENWYKA
>5LFZ-ArCE4
AGQPEPVATPPAVDCATTKCVALTFDDGPGEYTNRLLDELSEQHTPATFFVLGKNVKKYPKTLKRMVDEGHQIGSHTFDHKDITKLTA
EGIEHEVQWTDEAIEQAAGVKPQILRPPYGAHGAVYDRLIPYPLVLWDVDTLDWKHHDPQKTVRIALEEAKPGSIILMHDIHESSVKAV
PQLVSKLHDAGYTLVTVDQLFAGTDFKPAKAYDHRFKTNP
>5Z34
TELPLATPCDEEACKLPDCRCSSTNIPGGLRARDTPQFVTVTFDDGINVINIETYREVLYGRSNSNRCPAGATFYVSHEYTNYQLVNELY
NRGFEIALHSISHRTPQAFWADATYQNLVQEIGDQKRQMAHFASIPASAIKGVRIPFLQMSGNTSFQVMADFDLLYDCTWPTTALTNPG
LWPYTLHHESIQDCIIPPCPTASIPGPWVLPMISWRDLNNFPCSMVDGCFFTPDRTDEEGWFKFILTNFERHYLGNRAPFGFFVHEWFISS
NPAIKRAFVRFMDIINNLNDVFMVNSAEVIDWVKNPVPIDRYRQQQCKFTMPSICRPSFCGPLTGTHNQLSYYMTICNTCPRNYPWVGN
PLGQ
>A.nidulans CDA
MLFPNVPGVASLLSLFAATLILNTQALAINTIANLTDRQPRVSYGLYIHHCYVPGVVALTFDDGPYIYTEELLDILAQYGAKATFFVNGH
NLAGNEWLIQRVVNEGHQLASHTWGHTDLTVLSYDQIVDQMTRLESAFVASVGVVPTYMRPPYLAANDYVLGVMAELGYHVIGASV
DTKDYENDHPDLIGRSVAKFNQELDQGGTIVLSHDIHEQTVRTLTHIMLEEVYERGLQPTTVGGCLGDDAWYR
>R.circinans CDA1
MVWKTAIFSAIAIASVNAVTTNFTSKTDPTNITIPAIEQTTSHDPVKECTSYQSPYTINQAEWPTSWTTATSNGMNTSAEFQALYNSIDWT
KAPNFPVRKLSATTGGLDMTGYDTATDPACWWSSTTCKTPKTEGINADLYACPEPDVWGLTFDDGPNCSHNAFYDYLEQNKLKASMF
YIGSNVLNWPYGALRGVKDGHHIAGHTWSHQLMTTLTNQEILAELYFTQKAIKYVTGITPKYWRPAQGDIDDRVRWIATQLNLTAILW
NLDTDDWAAGTTPGITAETVNQNYEDFIKMGSNGTFANTGNVVLSHEINNMTMSFFMNHYEEIKKAYTHVLDVATCMNITNPYQETTI
TFPTFDQAINGASVSTSNGSSTSASAKSAGTSVQLNASLLVAALMGLMVLA
>R.circinans CDA2
MVWKTAIFSAVAIASVNAVTTNFTSTVDPANITIPAIDQTTSLDPVKECTSYQAPFAFDQKEWPTNWDVATSNGMNTSAEFQALYKSID
WTKAPNFPVRGISTTTGGLDMTGYDTAKDPACRWSSTNCKTPKTEGINADIYACPEPDVWGLTFDDGPNCSHNAFYDYLEQNKLKAS
MFYIGSNVVNWPYGAMRGIKDGHHIAGHTWSHKLMTTLTNQEVLAELYYTQKAIKYVTGTTPKYWRPAQGDIDDRVRWIATQLNLT
AILWNLDTDDWAAGTTPGITAETVSQNYEDYIKMGSNGTFANSGNIVLTHEINNMTMGFFLDHYEEIKKAYTHVLDVATCMNITNPYV
ETTVTFPTFEQAIGGASAFASNGFFNGTSPLSVAAKSAGAPIQLNGSLLVAALMGLLVFA
>R.circinans CDA3
MYIKTSAIAIALLQVACFAEAAKKTSKSSKSSKLDKPQDYWKSFKSLVDPNNITIADIPQTTSTNPSDECKWYEPPSNFVYDTKEWPNLW
EIATSNGMTKTSEFQALNKSIEWSKAPKIPVRKAGSDGSLDMTKYSDSDPDCWWSSSTCTKPKHKDINEDIYACPEPETWGLTYDDGPN
CSHNAFYDYLEQNKIKASMFYIGSNVVNWPYGAQRGVKAGHHIADHTWSHQLMTTLTNDEVLAELYYTQKAIKMVTGVTPLYWRPA
FGDVDDRVRWIATQLNLTTVLWNLDTDDWAAGSSKTLDEVKATYDSYVEMGSNGTFATSGQIVLTHEIDNTTMSLAMEYLPKIKAAY
KNVVDVATCMNITYPYQEHNVSFAPFGSAADESTATSTNATASSASASAAATSDEPGTTVIPLAANKAQIASAGIQVNPNSLVFAAFVA
AAYFF
>S.cerevisiae Cda2p

MRIQLNTIDLQCIIALSCLGQFVHAEANREDLKQIDFQFPVLERAATKTPFPDWLSAFTGLKEWPGLDPPYIPLDFIDFSQIPDYKEYDQN
HCDSVPRDSCSFDCHHCTEHDDVYTCSKLSQTFDDGPSASTTKLLDRLKHNSTFFNLGVNIVQHPDIYHRMQKEGHLIGSHTWSHVYLP
NVSNEKIIAQIEWSIWAMNATGNHTPKWFRPPYGGIDNRVRAITRQFGLQAVLWDHDTFDWSLLLNDSVITEQEILQNVINWNKSGSGL
ILEHDSTEKTVDLAIKINKLIGDDQSTVSHCVGGIDYIKEFLS

Poly-ß-1,6-GlcNAc deacetylases:
>AdIcaB-4WCJ
SSGLVPRGSHMESPRTPAGTHLQGDGLVVLCYHRVLPSSRYAISRREFAQQLDYLRQVGVRFVTPQEAEDYLAGRIHLPGKLVLVTFDD
GDLSVYRHAFPVLKKRKIPFLFFVIAGQVGRKWEGFSMCSWEQIKEMVASGLCVVGLHTYDLHYWDSQAKKPVFLLPGRERLFAEDT
ARGTACLKEHLGLKTRYFAYPYGFGTPTTDEILRTQGFSLVFTLRAKVNRPGDAPFVGRVLVTPDSWPQVAAWAQASP
>SeIcaB
MKPFKLIFISALMILIMTNATPISHLNAQANEENKKLKYEKNSALALNYHRVRKKDPLNDFISLLSGSKEIKNYSVTDQEFKSQIQWLKA
HDAKFLTLKEFIKYKEKGKFPKRSVWINFDDMDQTIYDNASPVLKKYHIPATGFLITNHIGSTNFHNLNLLSKKQLDEMYETGLWDFES
HTHDLHALKKGNKSKFLDSSQSVASKDIKKSEHYLNKNYPKNERALAYPYGLINDDRIKAMKKNGIQYGFTLQEKAVTPDADNYRIPRI
LVSNDAFETLIKEWDGFDEEK
>EcPgaB
MLRNGNKYLLMLVSIIMLTACISQSRTSFIPPQDRESLLAEQPWPHNGFVAISWHNVEDEAADQRFMSVRTSALREQFAWLRENGYQPV
SIAQIREAHRGGKPLPEKAVVLTFDDGYQSFYTRVFPILQAFQWPAVWAPVGSWVDTPADKQVKFGDELVDREYFATWQQVREVARS
RLVELASHTWNSHYGIQANATGSLLPVYVNRAYFTDHARYETAAEYRERIRLDAVKMTEYLRTKVEVNPHVFVWPYGEANGIAIEELK
KLGYDMFFTLESGLANASQLDSIPRVLIANNPSLKEFAQQIITVQEKSPQRIMHIDLDYVYDENLQQMDRNIDVLIQRVKDMQISTVYLQ
AFADPDGDGLVKEVWFPNRLLPMKADIFSRVAWQLRTRSGVNIYAWMPVLSWDLDPTLTRVKYLPTGEKKAQIHPEQYHRLSPFDDR
VRAQVGMLYEDLAGHAAFDGILFHDDALLSDYEDASAPAITAYQQAGFSGSLSEIRQNPEQFKQWARFKSRALTDFTLELSARVKAIRG
PHIKTARNIFALPVIQPESEAWFAQNYADFLKSYDWTAIMAMPYLEGVAEKSADQWLIQLTNQIKNIPQAKDKSILELQAQNWQKNGQ
HQAISSQQLAHWMSLLQLNGVKNYGYYPDNFLHNQPEIDLIRPEFSTAWYPKND
>BbBpsB
GSHMPDPDDGLTFRVLSMHDVRDNLRASFADMPDQFAIETRTLTDLFEWIRVKGFNPISMQQIIDSRAGVRPLPPRPILLTFDDGYASTY
TKVFPLLAAFNYPAVVAVVTSWTDAPAGTKIRLSPKIEVPHDFFMTWAQLREMAQSGLVELASHSHNLHRGVLANPQGNEQPAASSRQ
YLPASGRYENDAEYRARVRQDLKTSAHLIRHHTGVTIRSIVWPYGAHNRDTDQVAAEVGLNIGLTLQPGPNTPDVALTQIRRSLVDYEV
NVATVARAMR
>AaPgaB-4U10
MDRYGVLAYHSVVDDTAAKEEKQYFPQTISANLLISHFNWLKDNGYNVVSWQQIIDAENGKSTLPEKAVVLSFDDGYATMYNVIYPIL
KAYNYPAVFAPVSSWLDTPVNQLIPYANIKLPRNVFVTWDQVREMEQSGLVEIASHTDNLHHGVRANPAGSQLPAVVAPEYKNNRYE
SKTEYKNRLVQDFSRSSKSIQRQIGKKPRIMVWPYGQFNDVAIDAAKQSGMTHHFALGQKIINKIGDRYVGRLLIDTETGFSTIKNFLDG
VDDESKLMR
